# The Association of Physical Activity and Stress-induced Neurocognitive Impairments in Inhibitory Control in Children

**DOI:** 10.1177/24705470241261581

**Published:** 2024-06-11

**Authors:** Sebastian Ludyga, Manuel Hanke, Anja Schwarz, Rahel Leuenberger, Fabienne Bruggisser, Vera Nina Looser, Markus Gerber

**Affiliations:** 1 Department of Sport, Exercise and Health, 27209 University of Basel, Basel, Switzerland

**Keywords:** event-related potentials, Trier Social Stress Test, N200, PSW, fitness

## Abstract

**Background:**

Evaluation stress can impair inhibitory control, limiting the ability of children to perform cognitively. However, evidence on protective factors is lacking as stress-induced cognitive impairments are poorly understood. High physical activity has been related to better inhibitory control and has the potential to buffer the response to a stressor. We investigated the association of physical activity and stress-induced changes in inhibitory control as well as its underlying cognitive control processes (i.e., conflict monitoring and resolution).

**Method:**

Participants (10 to 13 y) with either low (*N *= 55) or high moderate-to-vigorous physical activity (*N *= 55) completed the Trier Social Stress Test for Children (TSST-C) and a control task in a randomized order. During both conditions, salivary cortisol was collected. Additionally, a computerized Stroop task was administered before and after the experimental conditions. The N200 and positive slow wave (PSW) components of event-related potentials elicited by the Stroop task were recorded using electroencephalography.

**Results:**

In comparison to the control task, the TSST-C elicited a pre-to post-test decrease of accuracy on incompatible trials. Path-analyses further revealed that this decrease was related to low physical activity and a reduced PSW amplitude. However, both the N200 and PSW amplitudes did not mediate the relation between physical activity groups and performance on the Stroop task.

**Conclusion:**

In children, evaluation stress decreases inhibitory control partly due to a reduced effectiveness of conflict resolution processes. Only children with high physical activity maintain inhibitory control after facing the stressor. However, this protective effect cannot be attributed to changes in conflict monitoring and resolution.

## Highlights

- Children with low physical activity showed a stress-induced decrease of inhibitory control
- In contrast, children with high physical activity maintained their cognitive performance despite the presence of evaluation stress
- Decreased effectiveness of conflict resolution partly explained lower inhibitory control following the stress task
- This process did not mediate the negative effect of low physical activity levels

## Introduction

Cross-national trend analysis has shown an increase in perceived schoolwork pressure along with psychosomatic health complaints among children in high income countries.^1^ A major source of this pressure is stress induced by goal structures that emphasize the avoidance of signs of incompetence (i.e., not making mistakes).^2^ This association is reciprocal, since examination stress can also have detrimental effects on performance determinants.^3^ In this respect, meta-analytical findings suggest that experimentally induced evaluation stress impairs higher order cognitive functions, including inhibitory control.^4^ This cognitive ability is necessary to override prepotent or dominant responses, to resist distractions, and to focus on the current task.^5^ As higher inhibitory control has been linked with reduced off-task behavior in the classroom^6^ and higher scores on academic tests,^7^ stress-induced impairments can affect the ability to learn and perform in school.

Understanding the mechanisms underlying such impairments can provide insights on protective factors and inform prevention strategies. Findings from event-related potentials elicited by cognitive tasks provide indications on the cognitive control processes affected by acute stress. In previous experiments with adults, successful stress induction led to alterations in conflict monitoring and detection,^8,9^ followed by impairments in the final stages of the inhibition processing stream.^10^ Despite the absence of stress-induced decrements in behavioral performance, these changes in response to cognitive tasks demanding response inhibition were still observed. When comparing the subdomains of inhibitory control, interference (i.e., resist stimulus conflict) seems to be more sensitive to acute stress than response inhibition (ie, resist response conflict).^4^ Consequently, interference tasks, such as the Stroop paradigm, might be more appropriate to investigate mechanisms underlying stress-induced performance impairments. This paradigm requires participants to respond to the color of ink of a color word, but to ignore its meaning (eg, “blue” written in green color). With regard to event-related potentials, the inhibition processing stream in the Stroop task involves two key processes: conflict monitoring indexed by a fronto-central N200 and conflict resolution indexed by a centro-parietal positive slow wave (PSW).^11^ The detection of the conflict and its subsequent handling are crucial for the adaptation of cognitive control on a trial-by-trial basis.^12^ A better ability to flexibly adjust these processes in response to conflict has been related to a lower cortisol reactivity and favorable stress adaptation.^13,14^ Consequently, the processes indexed by the N200 and PSW are vulnerable to acute stress, but at the same time they have a predictive value for the individual stress response.

Lifestyle factors that act on inhibitory control and its underlying mechanisms may have the potential to protect from or reduce the negative effects of acute stress on cognitive performance. In this respect, meta-analytical findings suggest that physical activity interventions in particular induce small benefits for a variety of cognitive abilities, including inhibitory control, in children.^15,16^ This may be explained by an altered neurocognitive profile as in previous experimental trials, improvements in children's behavioral performance were accompanied by greater effectiveness of conflict monitoring processes following physical activity interventions.^17,18^ Similarly, higher aerobic fitness has been related to more flexible upregulation of conflict monitoring on tasks with higher relative to lower inhibitory demands.^19,20^ Given that moderate-to-vigorous physical activity (MVPA) is related to aerobic fitness,^21^ this intensity range may be crucial for adaptations in conflict monitoring. The benefits of physical activity seem to extend beyond the early detection of conflict. For example, physical activity has also been linked to higher amplitudes of the P300,^22^ which indexes inhibition processes that are modality-unspecific and related to motor processing.^23^ The P300 and PSW components share common neural generators, including the prefrontal cortex and anterior cingulate cortex.^11,24,25^ The PSW might therefore also be characterized by a sensitivity to physical activity.

Recalling that acute stress can interfere at several stages within the inhibition processing stream, the specific neurocognitive profile promoted by physical activity may enable physically active children to maintain their inhibitory control when faced with a stressor. However, studies exploring the potential of physical activity as a protective factor are scarce. In young adults, evaluation stress induced by the Trier Social Stress Test led to an increase in salivary cortisol, but no changes in inhibitory control, irrespective of the individual physical activity level.^26^ This finding is contrasted by a less pronounced decrease in cognitive performance following successful stress induction in children with a high cortisol response and high physical activity.^27^ Due to the lack of a control condition, these results do not allow definite conclusions on the effect of acute stress on cognition. However, they provide indications that physical activity has the potential to counteract stress-related behavioral performance impairments in children in particular.

Addressing the lack of studies on the association between stress, cognition, and physical activity, we compared changes in inhibitory control following acute evaluation stress between children with high and low MVPA levels. We further examined whether event-related potential components indexing conflict monitoring and resolution (i.e., N200, PSW) underlie stress-induced changes in behavioral performance on the inhibitory control task. Based on previous findings,^17,18,27^ we expected children with higher levels of MVPA to show less pronounced impairments in inhibitory control and a mediation of this relation by N200 or PSW amplitudes.

## Methods

### Participants

Participants were recruited by distributing flyers containing study information at schools, sports clubs, and medical practices in Basel, Switzerland. Inclusion criteria were MVPA levels of either 30 min/d or less or 60 min/d or more, age 10 to 13 years, right-hand dominance, corrected-to or normal vision, and the ability to fully engage in physical activity within the last 3 months. The upper MVPA criterion was in accordance with the WHO recommendations.^28^ Both the lower and upper limits were chosen to include participants that show higher or lower MVPA levels compared to the average Swiss preadolescents.^29^ Conditions associated with an increased health risk during physical activity (e.g., acute infection, syncope, uncontrolled arrhythmia), attendance of special education services, sleep complaints, and the diagnosis of a psychological disorder served as exclusion criteria. Legal guardians of the participants provided written informed consent. The study protocol was approved by the local ethics committee (Ethikkommission Nordwest- und Zentralschweiz; EKNZ no. 2020-02622, Basel, Switzerland) and complied with the Declaration of Helsinki.

### Procedures

Screening involved the subjective recall of physical activity and objective accelerometer recordings. Those fulfilling the MVPA criterion were invited to complete an experimental and control condition distributed across two laboratory visits (separated by at least 4 days). The order of the conditions was randomized (permuted blocks) with MVPA (30 min/d or less; 60 min/day or more) used as stratum. SealedEnvelope™ (London, UK) created the randomization list and allocation was concealed. Additionally, participants and their legal guardians were blinded to the conditions.

Each laboratory visit included a familiarization with cognitive testing to reduce learning effects and to increase comfort. Twenty minutes after the familiarization, participants performed a Stroop task before and after the experimental and control conditions. During the cognitive task, event-related potentials were recorded via electroencephalography (EEG). Throughout the experimental and control sessions, salivary cortisol was assessed at regular intervals. Moreover, anthropometric data was collected and participants filled in the Pubertal Development Scale,^30^ Family Affluence Scale,^31^ Insomnia Severity Index,^32^ and Strengths and Difficulties Questionnaire.^33^

Assessments were scheduled in the afternoon (1 p.m to 5 p.m), with minimal variation in time of day between the two visits. Data collection took place in a dimly-lit room and at an environmental temperature between 20 and 22 °C, with surrounding noise kept to a minimum. Participants were instructed to avoid engagement in MVPA and intake of any medication within 24 h before the laboratory visit, to refrain from eating and drinking (except water) within 1 h before the appointment, and to avoid coming to the appointment in a rush.

### Experimental and Control Condition

In the experimental condition, participants underwent the standardized protocol of the Trier Social Stress Test for Children (TSST-C). This task encompasses an initial preparation phase (5 min), a free speaking phase that required participants to continue a story (5 min) and a mental arithmetic task with difficulty adjusted to age. The set-up included a camera recording the participant's performance and an evaluation committee of two experts, who were blinded to participants’ MVPA levels. The only deviation from the original TSST-C protocol was the use of face masks by those experts, which was required due to COVID-19 restrictions.

In the control condition, participants were asked to silently read a story (10 min) in a separate room. Subsequently, the experimenter engaged them into a conversation about the story and asked easy-to-answer questions. Similar to the TSST-C, the control task included a preparation phase and a story-related speech task, but performance pressure and socio-evaluative threat were minimized. To avoid anticipation effects, the TSST-C setting was hidden from participants until the task was administered.

### Physical Activity

During a regular school week (5 school days, 2 weekend days), participants wore triaxial accelerometers (ActiGraph wGT3X-BT, ActiGraph, Pensacola, FL, USA) on the hip. A valid dataset included at least six days with a total daily recording period of 10 h or more during daytime. Data digitized at a sampling rate of 50 Hz was processed off-line with ActiLife 6.13.4 (ActiGraph, Pensacola, FL, USA). Following wear time validation with the algorithm by Troiano et al,^34^ daily time spent in light, moderate, and vigorous intensity was calculated by applying an age-appropriate algorithm on counts per minute.^35^ Using a recall protocol, participants also reported their physical activities for the time period during which the accelerometer was worn. Non-wear time due to physical activities that could not be recorded with the device, such as swimming (because the accelerometer was not waterproof) and martial arts (due to an increased risk of injury), was replaced by data captured at the same time point in the recall protocols. Consequently, values provided are the sum of accelerometer-based MVPA and recall-based MVPA during non-wear time.

### Salivary Cortisol

Using Salivette® Blue cap (Sarstedt, Nümbrecht, Germany), saliva samples were collected 10 min and 40 min after arrival, directly before and after the TSST-C and control task as well as 15 min and 30 min after the task. Samples were stored at − 20 °C and were sent to the Biochemical Laboratory of the University of Trier, Germany, for analysis of cortisol concentrations. Within both conditions, the sample before the task was used as baseline and the area under the curve with respect to the increase (AUC_I_) was calculated.

### Cognitive Task

During the Stroop task, the German color words “blau” (blue), “grün” (green), and “gelb” (“yellow”) were presented against a black background on a computer screen. These words appeared in the same color of ink on compatible trials and in a different color of ink on incompatible trials. Participants were instructed to press a button corresponding to the meaning of the color word, independent of the color of ink it was written in. The trial procedure consisted of a fixation period of a random duration between 900 and 1100 ms, the presentation of the stimulus over 200 ms, and the subsequent 1100 ms response window. Following four examples and a practice round, a total of 192 trials were distributed equally across eight compatible and eight incompatible blocks. Both types of blocks alternated and the order of the color words were randomized. Feedback on the accuracy of the response was provided for practice trials only. For compatible and incompatible trials, average accuracy rates and reaction times (on response-correct trials only) were calculated.

### ERP Recording and Processing

A cap containing 32 active EEG electrodes (with 20 electrodes positioned in alignment with the 10:20 system) and 16 functional nearinfrared spectroscopy optodes was mounted to the participant's head. As the current study focused on event-related potentials, only the EEG methodology is described in further detail. Prior to recordings, impedances were controlled and reduced to 10 KΩ or lower by inserting highly conductive gel. The vertex channel served as online reference and the ground electrode was positioned at AFz. The actiCHamp (Brain Products GmbH, Gilching, Germany) amplified data recording and the sampling rate was set to 512 Hz. BrainVision Recorder 1.21 (BrainProducts GmbH, Gilching, Germany) was used for online data collection and BESA Research 7.1 (Brain Electric Source Analysis, Gräfelfing, Germany) for subsequent off-line processing.

In continuous raw data, blinks and eye movements were detected in virtual VEOG and HEOG channels and submitted to automatic adaptive artifact correction. Following principal component analysis, reconstructed artifact components from the data were subtracted using spatial filtering.^36^ Data was filtered (high-pass: forward phase shift of 0.3 Hz; slope 6 dB/octave; low-pass: zero-phase shift of 30 Hz; slope 24 dB/octave) and baseline-corrected using the period from −200 ms to stimulus onset as reference. Artifact rejection was performed using individual gradient and amplitude thresholds to remove artifacts that survived the initial correction procedure. Segments were created separately for trials with correct responses, both compatible and incompatible, ranging from −200 to 1000 ms relative to stimulus onset. Additionally, the reference was changed to average mastoids. The latency range used to extract ERP components was based on previous literature and, if necessary, adjusted based on visual inspection of the grand averaged data. Within 270 to 350 ms, the N200 was calculated as mean differences in amplitudes at fronto-central region (FC1, FC2, Fz) between incompatible and compatible trials. The PSW was extracted as mean difference between trial types within the 500 to 750 ms latency range at parietal region (P3, P4, Pz). Consequently, positive values denote more positive amplitudes on incompatible compared to compatible trials.

### Statistical Analysis

Statistical analyses were performed with SPSS 29.0 for Windows and the AMOS 29.0 plug-in (IBM, Armonk, NY, USA). Prior to main analyses, a one-way ANOVA was employed to investigate differences in anthropometrics, socio-economic status, pubertal status, sleep, and psychopathology between low and high MVPA groups. Zero-order correlations were calculated between these characteristics, sex, and changes in performance on the Stroop task and ERP components. For a manipulation check, salivary cortisol responses in the experimental and control session as well as differences in cognitive performance between pre- and post-test (in each condition) were compared with paired Student's *t*-Test.

The association of MVPA group and stress-induced changes in outcomes was examined using path analysis. The initial models predicted changes (post-test–pre-test) in accuracy and reaction time on incompatible (model 1a) or compatible (model 1b) trials of the Stroop task during the experimental session, while controlling for changes in the same set of outcomes during the control condition. Covariances between accuracy and reaction time within sessions were estimated to account for a potential speed accuracy trade-off. The subsequent models investigated indirect associations of MVPA group with behavioral performance on incompatible (model 2a) and compatible trials (model 2b) by including changes in N200 and PSW amplitudes during the stress condition, while controlling for changes in these ERP components during the control condition. Based on the results of zero-order correlations, background variables that correlated statistically significant with changes in one or more outcomes were accounted for in all models by covariance estimation. For all analyses, three-way interactions were decomposed using Bonferroni-corrected post hoc tests. The level of statistical significance was set to *P *≤ .05. Model fit was tested and considered appropriate at χ2/df ≤ 2 and root mean square error of approximation (RMSEA) ≤ .08.^37,38^

## Results

Data was collected from 110 participants. The high MVPA group (*N* = 34 f / 21 m) was characterized by higher MVPA, *P *< .001, η^2 ^= 0.761, lower stress, *P *= .002, η^2 ^= 0.087, lower psychopathology, *P *= .045, η^2 ^= 0.037, earlier pubertal stage, *P *< 0.001, η^2 ^= 0.098, and higher socio-economic status, *P *= .008, η^2 ^= 0.064, compared to the low MVPA (*N* = 14 f / 41 m) group (Table 1). With the exception of sex, baseline characteristics were not correlated with changes in cognitive performance outcomes. Therefore, only sex was considered as covariate in main analysis due to its correlation with changes in accuracy, *r *= −0.24, *P *= .012, and reaction time on incompatible trials, *r *= −0.22, *P *= .019, in the stress condition. An overview of mean reaction times and accuracy rates on the Stroop task as well as amplitudes of N200 and PSW at pre- and post-test is provided in Supplementary Table 1 and 2, respectively.

**Table 1.** Participants’ characteristics.

	Low MVPA (*N* = 55)		High MVPA (*N* = 55)		F
	M	SD	M	SD	
Age in y	12.2	1.1	11.9	1.1	1.31
MVPA in min^.^d^−1^	25.3	9.0	71.1	16.0	343.80*
Weight in kg	45.2	11.9	41.3	9.1	3.65
Body mass index in kg^.^m^−2^	18.3	3.3	17.4	2.3	2.95
Perceived stress scale	16.1	6.1	12.6	5.5	10.25*
Pubertal development scale	6.2	2.6	4.6	2.0	11.78*
Family affluence scale	6.7	1.6	7.4	1.3	7.39*
Insomnia severity index	3.5	2.8	3.9	3.3	0.44
SDQ score	10.2	4.7	8.4	4.7	4.11*

*Notes:* * *P* < .05; Abbreviations: MVPA: moderate-to-vigorous physical activity; SDQ: Strengths and Difficulties Questionnaire.

### Stress Induction

With regard to the experimental manipulation, the stress induction was successful as participants’ salivary cortisol (AUC_I_) was higher in the stress (68.8 ± 111.2 nmol/l) compared to the control condition (26.0 ± 79.7 nmol/l), T = 3.49, *P *< .001, *d *= 0.33. Similarly, accuracy on incompatible trials decreased from pre- (85.6 ± 10.2%) to post-test (83.6 ± 11.1%) in the stress condition, *T *= 2.86, *P *= .005, *d *= 0.27, whereas no difference between pre- (84.9 ± 10.4%) and post-test (83.9 ± 11.0%) was found in the control condition, T = 1.33, *P *= .188, *d *= 0.13.

### Cognitive Performance

**Figure 1.**
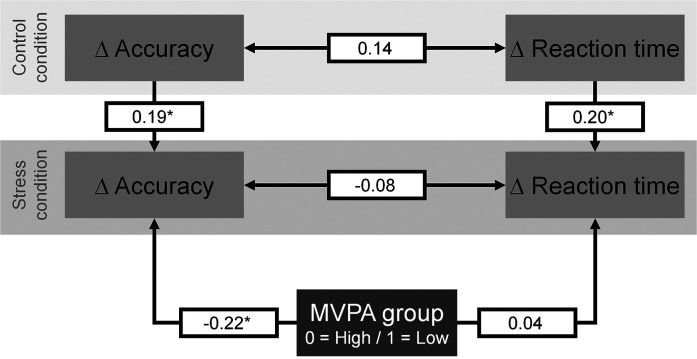
Association of the low and high moderate-to-vigorous physical activity groups with changes in accuracy from pre- to post-test on incompatible trials during the stress condition.

*Notes:* * *P* < .05; MVPA: moderate-to-vigorous physical activity. Positive change scores denote an increase and negative change scores denote a decrease from pre- to post-test within the experimental conditions. Model is controlled for sex. Standardized regression coefficients are shown.

**Figure 2.**
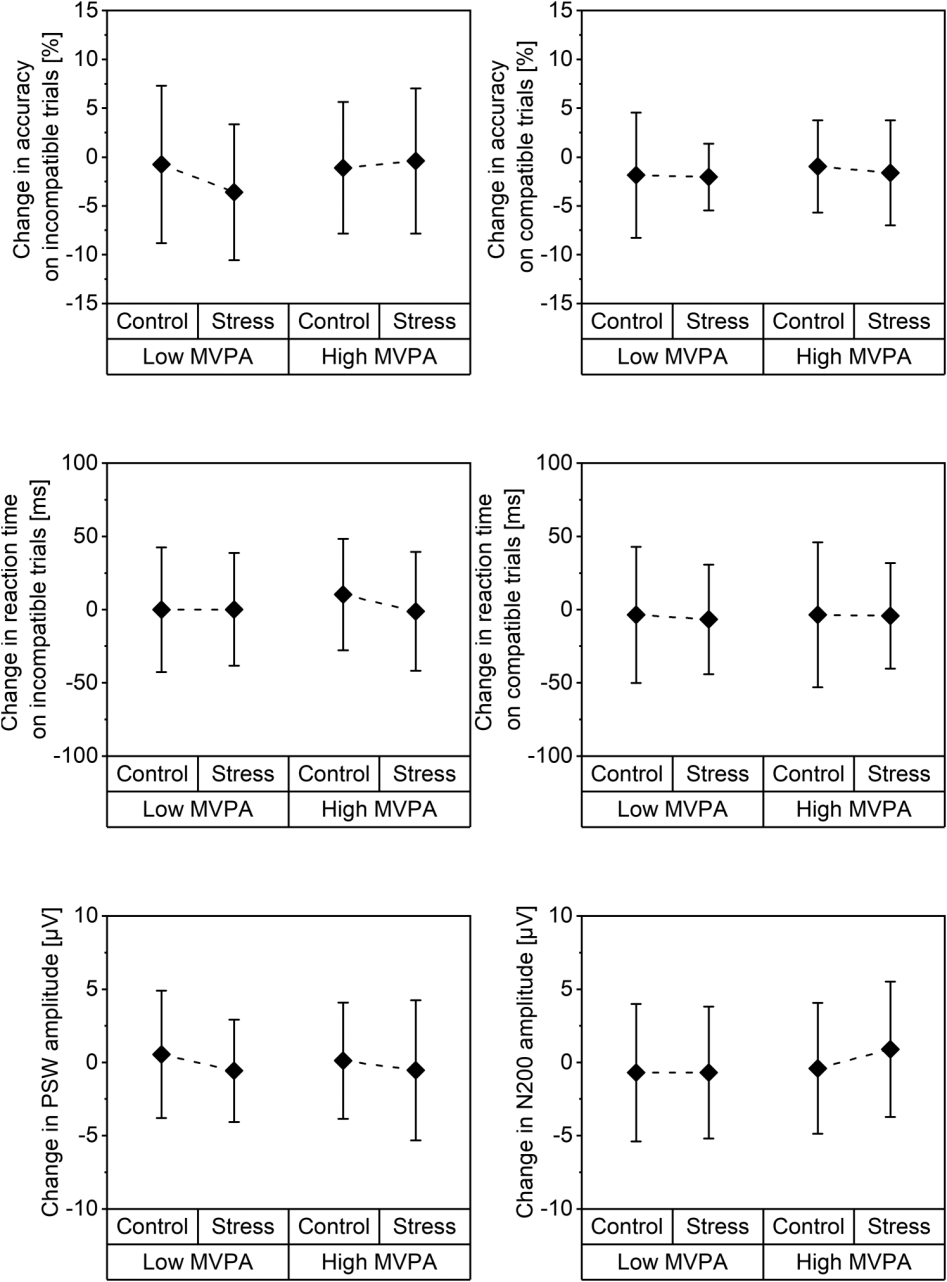
Moderate-to-vigorous physical activity group differences in pre- to post-test changes (means and standard deviation) in accuracy (upper row) and reaction time (middle row) on the Stroop task as well as changes in event-related potential components (lower row) across the stress and control condition.

*Notes:* Positive values denote an increase and negative values denote a decrease from pre- to post-test within the experimental conditions. MVPA: moderate-to-vigorous intensity physical activity.

For performance on incompatible trials (model 1a), path analysis showed that group was inversely associated with change in accuracy during the stress condition (Figure 1), β = −0.22, *P *= .015, indicating a more pronounced decrease from pre- to post-test in the low MVPA group (Figure 2). In contrast, group was not related to stress-induced changes in reaction time. For both accuracy, β = 0.19, *P *= .031, and reaction time, β = 0.20, *P *= .035, higher changes in the control condition were associated with higher changes in the stress condition.

On compatible trials, there was no association of group with changes in accuracy, β = −0.07, *P *= .487, and reaction time, β = −0.03, *P *= .727, during the stress condition (Supplementary Figure 1). The fits of the models 1a, Χ^2^/df = 0.62; RMSEA < 0.001, and 1b, Χ^2^/df = 0.21; RMSEA < 0.001, to the data were appropriate.

### Event-related Potentials

**Figure 3.**
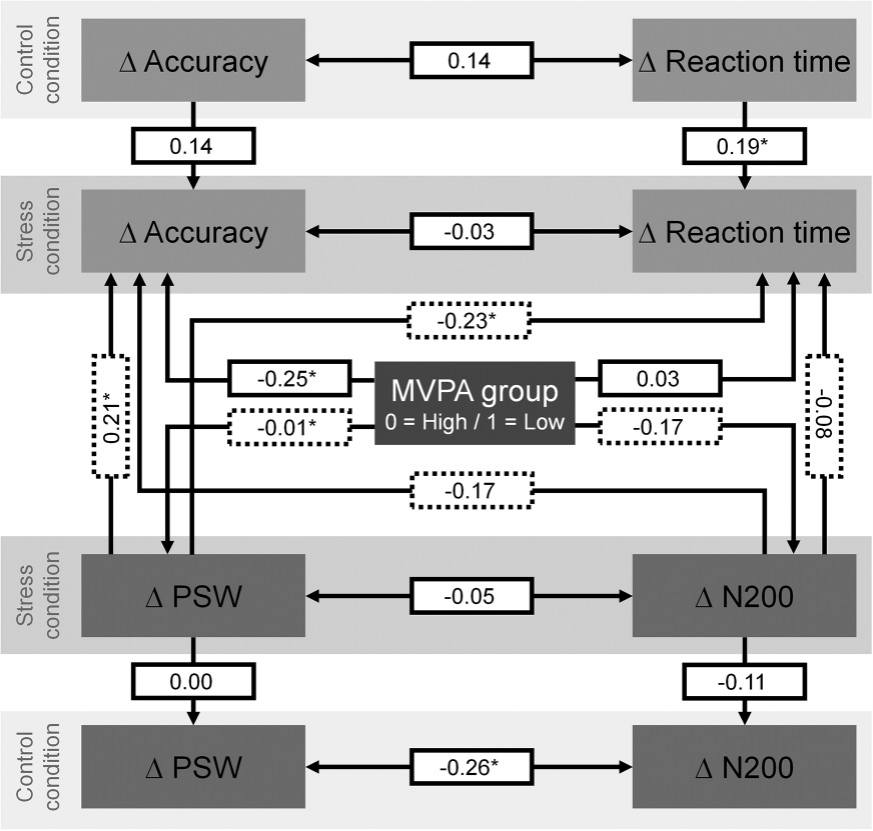
Association of the low and high moderate-to-vigorous physical activity groups with pre- to post-test changes in accuracy on incompatible trials during the stress condition and the mediation of this association by PSW and N200 amplitudes.

*Notes:* * *P* < .05; MVPA: moderate-to-vigorous physical activity. Positive change scores denote an increase and negative change scores denote a decrease from pre- to post-test within the experimental conditions. Model is controlled for sex. Standardized regression coefficients are shown. PSW: positive slow wave; MVPA: moderate-to-vigorous physical activity.

When ERP components were introduced (model 2a), the association of the low MVPA group with changes towards decreased accuracy on incompatible trials during the stress condition remained statistically significant (Figure 3), β = −0.25, *P *= .005. Consequently, no mediation of this association by ERP components was indicated. However, an increase in PSW amplitude from pre- to post-test was related to an increase in accuracy, β = 0.21, *P *= .016, and decrease in reaction time, β = −0.23, *P *= .010, on incompatible trials during the stress condition (Figure 2). Additionally, the association between increasing N200 amplitude with decreasing accuracy on incompatible trials approached the level of statistical significance, β = −0.17, *P *= .067. 

**Figure 4.**
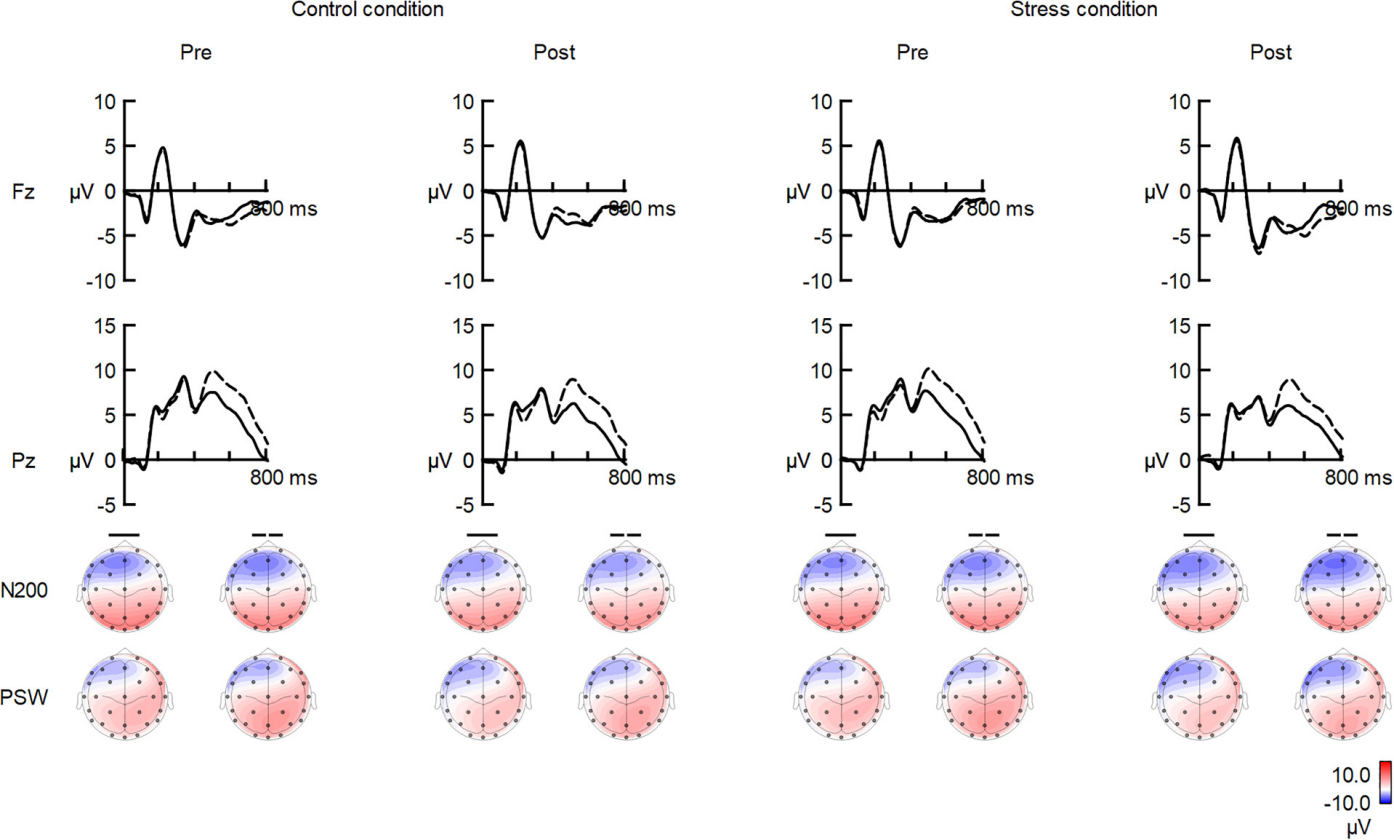
Event-related potential waveforms elicited by the Stroop Color-Word task in the control and stress conditions at pre- and post-test displayed at Fz and Pz.

*Notes:* Waveforms are collapsed across all participants. Solid lines represent compatible trials, whereas dashed lines represent incompatible trials. Topographic plots are shown for N200 (270 to 350 ms) as well as the PSW (500 to 750 ms). PSW: positive slow wave

For performance on compatible trials, there was no association between MVPA group and changes in reaction time as well as accuracy during the stress condition, when ERP components were included as mediators (Supplementary Figure 2). Among the mediators, only changes in PSW amplitude were related to increased accuracy on compatible trials during the stress condition, β = 0.23, *P *= .013. The model fits of 2a, Χ^2^/df = 1.50; RMSEA = 0.068, and 2b, Χ^2^/df = 1.42; RMSEA = 0.062, were appropriate. Waveforms for the event-related potentials and the topographic dsitribution of N200 and PSW amplitudes are shown in Figure 4.

## Discussion

Our study compared children with high and low MVPA levels with regard to changes in behavioral and neuroelectric indices of inhibitory control following acute evaluation stress. Following the successful stress induction as indicated by increased salivary cortisol, a decrease in accuracy on incompatible trials of the Stroop task was found in the low MVPA group. In contrast, the high MVPA group maintained their behavioral performance levels throughout the TSST-C. Stress-induced changes in accuracy on incompatible trials were partly explained by altered conflict resolution. However, this cognitive control process did not mediate the association between MVPA group and changes in inhibitory control following the TSST-C.

Our findings show that the detrimental effects of evaluation stress observed for working memory in children^27^ also extend to inhibitory control and interference in particular. This effect was observed from reduced accuracy rates on incompatible trials of the Stroop task, while reaction times remained unchanged. Consequently, there was no speed accuracy trade-off, meaning that the TSST-C led to a decreased behavioral performance rather than a strategy change from prevention to promotion focus. The reduction in accuracy rate was linked to a decrease in PSW amplitude and a trend towards decreasing negativity of the N200 amplitude. As both ERP components were calculated from difference waves, the detrimental effect of stress on performance was indexed by a reduction of amplitude differences between compatible and incompatible trials. Based on the functional significance of the PSW,^25^ this pattern indicates a limitation of the ability to adapt or upregulate cognitive resources needed to resolve the stimulus conflict induced by incompatible trials. Our results further provide some indications that the TSST-C might have affected accuracy on the Stroop task by a decreased effectiveness of conflict monitoring, which was indexed by a trend towards lower negativity of the N200 amplitude.^11^ Both the N200 and PSW contribute to post-conflict adjustments in cognitive control,^12^ and stress-induced decreases in amplitudes may be a compensatory mechanism to reallocate resources to deal with the stressor. This is further supported by findings showing that the prefrontal cortex, which serves a main neural generator of the PSW,^25^ is sensitive to stress. In this respect, prefrontal networks seem to weaken in response to a stressor, triggering a change from reflective (ie, informed strategic decisions) to reflexive (ie, fast decisions) control of behavior.^39^

Even though decreased effectiveness of conflict resolution partly explained lower inhibitory control following the stress task, this process did not mediate the negative effect of low physical activity levels. In other words, the maintenance of performance on incompatible trials across both the stress and control conditions in participants with higher physical activity does not seem to be due to an influence on processes within the inhibition processing stream. The pattern of results might instead be explained by compensatory mechanisms of the autonomous nervous system. Increased parasympathetic activity in the resting state and in response to a stressor have both been related to better inhibitory control 30 min following evaluation stress in young adults.^40^ Similarly, adapting parasympathetic activity to Stroop tasks that vary in socio-emotional demands has predicted greater stability in behavioral performance.^41^ In a recent review, this vagal flexibility was discussed as an index of self-regulatory abilities, which are required to adapt cognitive processes, including inhibitory control, to varying environmental demands.^42^ Physical activity seems to buffer physiological responses to stress that are linked with a withdrawal of parasympathetic activity.^43^ Examples include a lower reactivity of heart rate, blood pressure, and cortisol.^44–46^ Moreover, physical activity has been associated with generally increased parasympathetic activity in children.^47^ Despite less effective conflict resolution, children with high physical activity could have maintained inhibitory control during the stress condition due to the unique contribution of higher vagal flexibility to behavioral performance. This maintenance would not simply be due to lower levels of stress reported in the high MVPA group as initial correlation analyses provided no support for an association between perceived stress and change in inhibitory control during the experimental conditions.

Our study revealed novel insights into children's responses to evaluation stress and the potential of physical activity to buffer negative effects on inhibitory control. Due to the comparison of groups differing in physical activity, it remains unclear whether the protective effect mainly results from the specific profile and traits of physically active children or whether an increase in physical activity can elicit same effects. An understanding of the underlying mechanisms is necessary for causal inferences. We recommend that future studies specifically examine changes in the autonomic nervous system as the protective effect does not seem to originate from alterations in the inhibitory processing stream. Another limitation of our study is its focus on aspects of inhibitory control that are measurable through the Stroop task. Thus, the role of physical activity as a protective factor and the effects of evaluation stress may not necessarily generalize to other components of executive functions. From a methodological perspective, the use of the TSST-C, which induces evaluation stress in a laboratory setting, further limits the generalization of our findings. Although ecological validity of this task has been proved,^48^ it is unclear whether high physical activity also protects from stress-induced performance impairments in a more naturalistic task and real-life setting. Lastly, there was an unequal distribution of boys and girls between the MVPA groups. This pattern accords well with previous data showing that the proportion of boys accumulating 60 min MVPA per day is much higher than girls and may be representative for Swiss preadolescents. However, we did not address whether sex actually moderates the association of MVPA levels with stress-induced changes in inhibitory control.

## Conclusion

In children, evaluation stress induces an increase in cortisol concentration and reduces inhibitory control. At a neurocognitive level, decreased effectiveness of conflict resolution and post-conflict adaptation partly account for this. Consequently, behavioral strategies that ensure the effectiveness of cognitive control processes in the presence of an evaluative stressor have the potential to counteract temporary deterioration in cognitive performance. Although high physical activity levels are associated with the maintenance of inhibitory control under evaluation stress, the protective effect of physical activity is not mediated by conflict monitoring and resolution processes.

## Supplemental Material

sj-docx-1-css-10.1177_24705470241261581 - Supplemental material for The Association of Physical Activity and Stress-induced Neurocognitive Impairments in Inhibitory Control in Children Supplemental material, sj-docx-1-css-10.1177_24705470241261581 for The Association of Physical Activity and Stress-induced Neurocognitive Impairments in Inhibitory Control in Children by Sebastian Ludyga, Manuel Hanke, Anja Schwarz, Rahel Leuenberger, Fabienne Bruggisser, Vera Nina Looser and Markus Gerber in Chronic Stress
