# Supplementary material for: The Association of Physical Activity and Stress-induced Neurocognitive Impairments in Inhibitory Control in Children
Source: Chronic Stress (Thousand Oaks). 2024 Jun 11;8:24705470241261581. doi: 10.1177/24705470241261581 (PMC11168053; doi:10.1177/24705470241261581)
Supplement: sj-docx-1-css-10.1177_24705470241261581 - Supplemental material for The Association of Physical Activity and Stress-induced Neurocognitive Impairments in Inhibitory Control in Children [file sj-docx-1-css-10.1177_24705470241261581.docx]

**SUPPLEMENT**

**Supplementary Figure 1.** Association between the low and high moderate-to-vigorous physical activity groups with changes in accuracy from pretest to posttest on compatible trials during the stress condition.

**
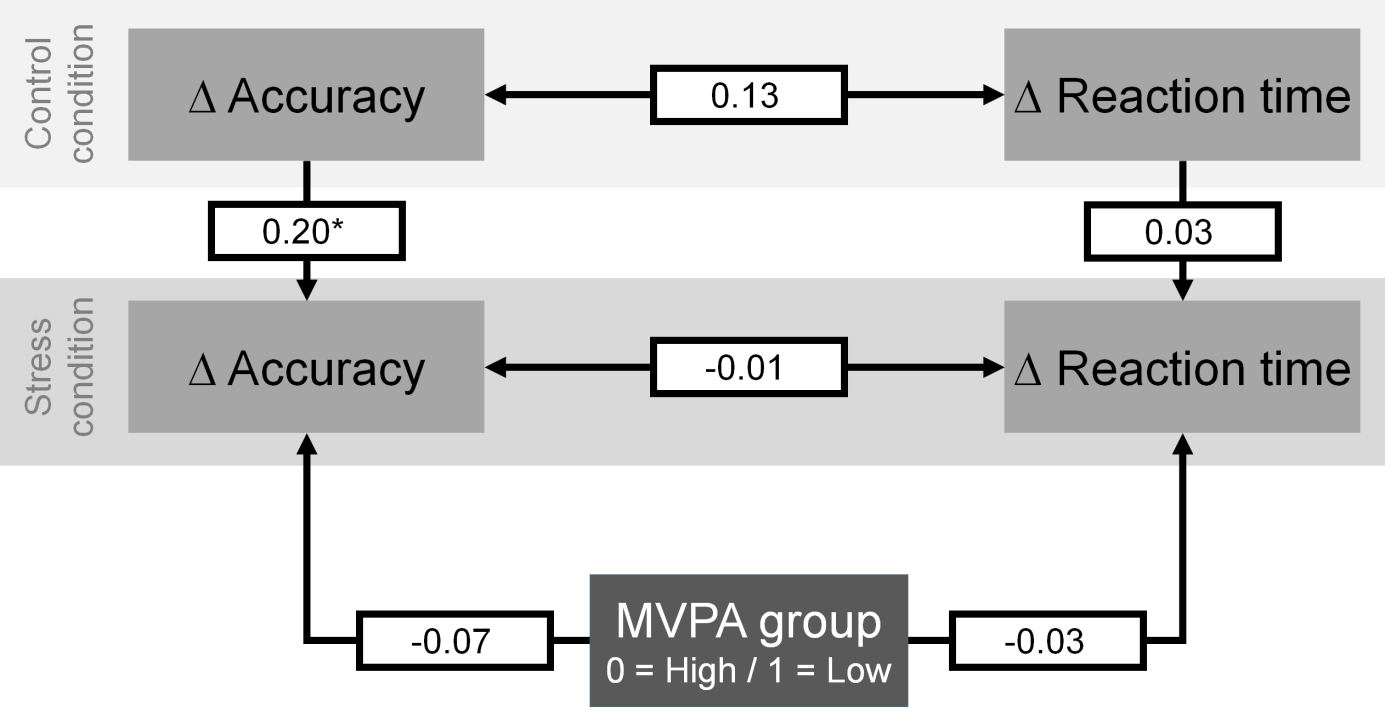
**

*Notes:* * p < 0.05; MVPA = Moderate-to-vigorous physical activity. Positive change scores denote an increase and negative change scores denote a decrease from pre- to posttest within the experimental conditions. Model is controlled for sex. Standardized regression coefficients are shown.

**Supplementary Figure 2.** Association of the low and high moderate-to-vigorous physical activity groups with pre- to posttest changes in accuracy on compatible trials during the stress condition and the mediation of this association by PSW and N200 amplitudes.

**
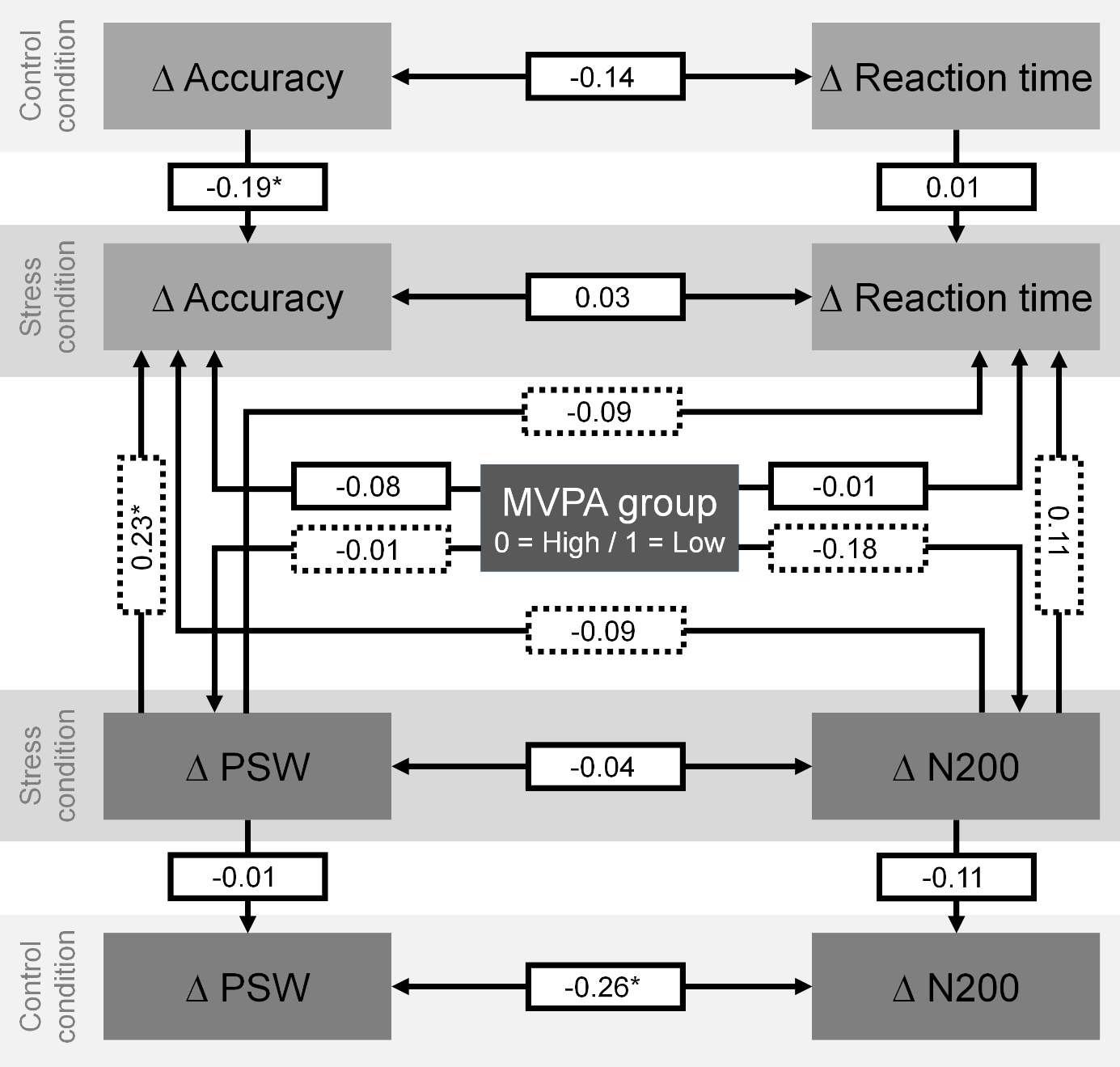
**

*Notes:* * p < 0.05; MVPA = Moderate-to-vigorous physical activity. Positive change scores denote an increase and negative change scores denote a decrease from pre- to posttest within the experimental conditions. Model is controlled for sex. Standardized regression coefficients are shown.

**Supplementary Table 1.** Comparison of cognitive performance at pre- and posttest in the control and stress condition between moderate-to-vigorous physical activity groups.

|  | Condition | Trial | Low MVPA (*N = 55*) | | | | High MVPA (*N = 55*) | | | |
| --- | --- | --- | --- | --- | --- | --- | --- | --- | --- | --- |
|  |  |  | Pre | | Post | | Pre | | Post | |
|  |  |  | *M* | *SD* | *M* | *SD* | *M* | *SD* | *M* | *SD* |
| Reaction  time in ms | Control | Incompatible | 691.1 | 91.2 | 691.1 | 96.4 | 698.8 | 90.9 | 688.6 | 101.0 |
|  |  | Compatible | 602.8 | 90.4 | 606.5 | 90.1 | 607.8 | 84.4 | 611.3 | 96.2 |
|  | Stress | Incompatible | 690.4 | 94.8 | 690.3 | 95.4 | 699.8 | 95.7 | 700.9 | 91.4 |
|  |  | Compatible | 602.2 | 91.3 | 608.9 | 89.5 | 612.7 | 89.4 | 616.9 | 91.7 |
| Accuracy in % | Control | Incompatible | 85.0 | 10.6 | 84.2 | 10.2 | 84.7 | 10.3 | 83.6 | 11.8 |
|  |  | Compatible | 93.2 | 5.7 | 91.4 | 7.7 | 93.2 | 5.5 | 92.2 | 6.2 |
|  | Stress | Incompatible | 87.6 | 8.2 | 84.0 | 10.9 | 83.7 | 11.6 | 83.3 | 11.3 |
|  |  | Compatible | 94.1 | 4.6 | 92.1 | 5.6 | 92.6 | 6.2 | 91.0 | 7.5 |

*Notes:* * p < 0.05; MVPA = Moderate-to-vigorous physical activity.

**Supplementary Table 2.** Comparison of PSW and N200 amplitudes at pre- and posttest in the control and stress condition between MVPA groups.

|  | Condition | Low MVPA (*N = 55*) | | | | High MVPA (*N = 55*) | | | |
| --- | --- | --- | --- | --- | --- | --- | --- | --- | --- |
|  |  | Pre | | Post | | Pre | | Post | |
|  |  | *M* | *SD* | *M* | *SD* | *M* | *SD* | *M* | *SD* |
| PSW in µV | Control | 1.42 | 3.14 | 1.97 | 3.89 | 2.47 | 3.36 | 2.50 | 3.11 |
|  | Stress | 2.13 | 2.63 | 1.45 | 3.85 | 2.41 | 3.80 | 1.87 | 2.82 |
| N200 in µV | Control | 0.76 | 3.01 | 0.06 | 3.45 | 0.32 | 3.76 | -0.09 | 3.31 |
|  | Stress | 0.71 | 3.39 | 0.07 | 3.25 | -0.12 | 3.54 | 0.78 | 3.27 |

*Notes:* MVPA = Moderate-to-vigorous physical activity.
